# Evaluation of Gold Complexes to Address Bacterial Resistance, Quorum Sensing, Biofilm Formation, and Their Antiviral Properties against Bacteriophages

**DOI:** 10.3390/toxics11110879

**Published:** 2023-10-26

**Authors:** Ana Marques, Sónia A. C. Carabineiro, Manuel Aureliano, Leonor Faleiro

**Affiliations:** 1 Faculdade de Ciências e Tecnologia, Universidade do Algarve, Campus de Gambelas, 8005-139 Faro, Portugal; a54358@ualg.pt; 2 Algarve Biomedical Center—Research Institute, 8005-139 Faro, Portugal; 3 LAQV-REQUIMTE, Department of Chemistry, NOVA School of Science and Technology, Universidade NOVA de Lisboa, 2829-516 Caparica, Portugal; sonia.carabineiro@fct.unl.pt; 4 Centro de Ciências do Mar (CCMar), Universidade do Algarve, 8005-139 Faro, Portugal; 5 Champalimaud Research Program, Champalimaud Centre for the Unknown, 1400-038 Lisbon, Portugal

**Keywords:** gold complexes, chlorotrimethylphosphinegold (I), chlorotriphenylphosphinegold (I), dichloro (2-pyridinecarboxylate) Au (III), 1,3-bis(2,6-diisopropylphenyl) imidazole-2-ylidene Au(I) chloride, antibacterial, antibiotic resistance, antibiofilm, anti-quorum sensing, antiviral

## Abstract

The worldwide increase in antibiotic resistance poses a significant challenge, and researchers are diligently seeking new drugs to combat infections and prevent bacterial pathogens from developing resistance. Gold (I and III) complexes are suitable for this purpose. In this study, we tested four gold (I and III) complexes, (**1**) chlorotrimethylphosphine gold(I); (**2**) chlorotriphenylphosphine gold(I); (**3**) dichloro(2-pyridinecarboxylate) gold (III); and (**4**) 1,3-bis(2,6-diisopropylphenyl)imidazole-2-ylidene gold(I) chloride, for their antibacterial, antibiofilm, antiviral, and anti-quorum sensing activities. Results reveal that **1** significantly inhibits *Escherichia coli* DSM 1077 and *Staphylococcus aureus* ATCC 6538, while **2**, **3**, and **4** only inhibit *S. aureus* ATCC 6538. The minimum inhibitory concentration (MIC) of **1** for *S. aureus* ATCC 6538 is 0.59 μg/mL (1.91 μM), and for methicillin-resistant *S. aureus* strains MRSA 12 and MRSA 15, it is 1.16 μg/mL (3.75 μM). For *E. coli* DSM 1077 (Gram-negative), the MIC is 4.63 μg/mL (15 μM), and for multi-resistant *E. coli* I731940778-1, it is 9.25 μg/mL (30 μM). Complex **1** also disrupts biofilm formation in *E. coli* and *S. aureus* after 6 h or 24 h exposure. Moreover, **1** and 2 inhibit the replication of two enterobacteria phages. Anti-quorum sensing potential still requires further clarification. These findings highlight the potential of gold complexes as effective agents to combat bacterial and viral infections.

## 1. Introduction

Presently, one of the most significant threats to human, animal, and environmental health is the development of antibiotic resistance (AR) by pathogenic, commensal, and environmental bacteria. This critical problem has been triggered by the inappropriate overuse of antibiotics in both human and veterinary medicine, jeopardizing the effective treatment of both common and severe bacterial infections [1]. Moreover, bacterial pathogens can display tolerance responses to different hurdles, contributing to overcoming the exposure to antibiotics [2,3,4]. In 2019, six bacterial pathogens were individually responsible for more than 250,000 deaths related to AR, namely *Escherichia coli*, *Staphylococcus aureus*, *Klebsiella pneumoniae*, *Acinetobacter baumannii*, and *Pseudomonas aeruginosa* [1]. In the same year, methicillin-resistant *S. aureus* (MRSA) was responsible for more than 100,000 deaths and 3.5 million disability-adjusted life years (DALYs) when considering pathogen-drug combinations [1]. If the alarming current rate of AR increase continues, projections suggest that by the year 2050, approximately 10 million people per year could die due to AR-related infections [1,5].

The action of antibiotics against bacteria can be divided into five general mechanisms, which target essential physiological structures and processes: (1) cell wall synthesis, (2) protein synthesis, (3) DNA synthesis, (4) cellular membrane synthesis and integrity, and 5) folic acid metabolism. However, bacteria can survive antibiotic exposure via different mechanisms, which include (i) inactivation or modification of antibiotics, (ii) overproduction of the target, (iii) active efflux pump of the antibiotic, and (iv) diminished antibiotic uptake by decreased cell wall permeability [6,7]. Other factors, such as biofilm formation by bacterial cells, also contribute to AR [8] and are implicated in the persistence of pathogenic bacteria during chronic infections in clinical settings [9]. The ability to form biofilms is common among microorganisms and occurs via a sequence of five stages, namely (1) initial adhesion of planktonic cells (in suspension), during which the attachment of cells to an abiotic or biotic surface can be reversible, (2) permanent adhesion, which involves the production of extracellular polymeric substances (such as polysaccharides, proteins, extracellular DNA, and others), creating the biofilm matrix that provides a spatial structure, (3) development of microcolonies, (4) biofilm maturation, and (5) dispersal [10,11]. Biofilm formation, along with the production of virulence factors, toxin production, capacity for external DNA capture, secondary metabolite production, and bioluminescence, are coordinated activities governed by the chemical cell–cell communication system, known as quorum sensing (QS).

The QS system works in coordination with cell density, allowing a rapid and successful adaptation of the entire population to dynamic environmental changes via the production and detection of extracellular signaling molecules known as autoinducers [12]. The QS works in both Gram-negative and Gram-positive bacteria, but with the participation of different molecules, namely N-acyl-homoserine lactone (AHL), that is mainly produced by Gram-negative bacteria as their signaling molecule, whereas the signaling molecules produced by Gram-positive bacteria are peptides, such as auto-inducing peptides (AIP). Nevertheless, there are signaling molecules produced by both Gram-negative and Gram-positive bacteria, such as furanose borates (AI-2) and indole [13,14]. In the fight against antibiotic resistance and biofilms, research has explored the use of compounds capable of inhibiting QS, including metal complexes [15,16].

Gold complexes are well-known for their antibacterial activity, even against resistant strains [17,18,19,20]. They have the ability to inhibit essential enzyme activity, such as thioredoxin reductase [18,21]. While gold ions have been explored to combat biofilm formation, their application is still limited [20,22,23]. For example, Radzig et al. [22] showed that AuHCl_4_ was effective in inhibiting biofilm formation at different concentrations: nearly inhibiting *E. coli* AB1157 at 0.64 μg/mL, *Serratia proteamaculans* 94 at 10 μg/mL, and requiring the highest concentration of AuHCl_4_ to control biofilm formation of *P. aeruginosa* PAO1. At 150 μg/mL, AuHCl_4_ was able to eliminate sessile cells of *E. coli* AB1157. The antibiofilm activity of gold complexes was also reported in the study of Ratia et al. [20], where gold (III) complex **2**, stabilized in the form of C^S-cycloaurated, holding a diphenylphosphinothioic amide fraction, effectively inhibited the biofilm formation of two MRSA strains and two strains of *S. epidermidis* at concentrations ranging from 8 to 16 mg/L. Notably, the anti-quorum sensing activity of gold complexes has not been reported so far, to the best of our knowledge.

The therapeutic applications of gold have been well known for many centuries [24]. Gold compounds, such as sodium aurothiopropanol sulfonate, known under the name of allochrysine, and other compounds have been used to treat pulmonary tuberculosis and were also used for the treatment of rheumatoid arthritis by 1930 [25]. More recently, gold nanoparticles (AuNP) are also used for the treatment of rheumatoid arthritis as they offer beneficial efficacy and reduce toxic effects [26]. Studies revealed no major safety concerns with the use of AuNPs [27]. Still, the conversion of AuNP to clinical applications remains to be determined due to certain limitations and a lack of understanding of the adverse effects of long-term gold accumulation [26,27]. Gold has also been used safely in medicine since early times [28] and, more recently, also in vaccines [29,30]. The most commonly used gold compounds contain Au(I) and Au (III) [23,31]. Our research group found that certain gold complexes (I and III) strongly inhibit the activity of the sarcoplasmic reticulum Ca^2+^-ATPase, SERCA (sarco (endo) reticulum calcium ATPase), with IC_50_ values of 0.8 and 0.9 μM, respectively [32]. For other complexes, the IC_50_ values were higher (4.5 μM and 16.3 μM). These results show that in addition to Au(I) complexes, Au(III) compounds can act as inhibitors of P-type ATPases, possibly affecting two types of Ca^2+^-ATPases, including those obtained from PMCA (plasma membrane calcium ATPase and SERCA [31,32]. In addition to our investigations on gold compounds, our research group has been studying the mechanisms of action of other inorganic compounds, such as polyoxometalates (POMs) [33]. Our study showed that the POM-type Preyssler (P_5_W_30_) not only inhibits bacterial growth but also interferes with the quorum sensing system, preventing the formation of biofilms by antibiotic-resistant bacteria. Consequently, it hinders the process of bacterial colonization, the development of antibiotic resistance, and persistence, both in the environment and in the host. Furthermore, the P_5_W_30_ compound was found to reduce the replication capacity of enteric viruses [34]. Additionally, we observed that changes in lipid peroxidation may be involved in the biomedical action of POVs (polyoxovanadates) [35,36].

These studies demonstrate the potential applications of metal compounds and complexes in biomedicine, suggesting therapeutic opportunities and reinforcing the potential use of these metallopharmaceuticals in the near future.

The main objective of the present study was to further explore the biological activities of four gold (I) and (III) complexes, namely evaluate their antibacterial, antibiofilm, anti-quorum sensing, and antiviral properties, with the ultimate goal of contributing to the development of gold compounds as potential agents to combat bacterial infections.

## 2. Materials and Methods

### 2.1. Gold Compounds

**Figure 1:**
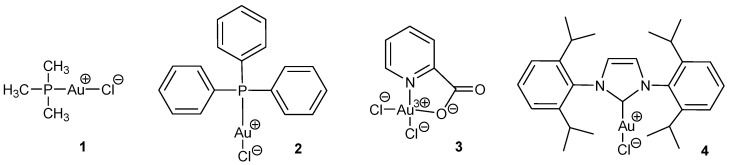
Structures of the gold complexes used in the study: (**1**) chlorotrimethylphosphine gold(I); (**2**) chlorotriphenylphosphine gold(I); (**3**) dichloro (2-pyridinecarboxylate) gold (III); and (**4**) 1,3-bis(2,6-diisopropylphenyl) imidazole-2-ylidene gold(I) chloride. Formal charge distribution is also shown.

Four gold (I) complexes were tested: (1) chlorotrimethylphosphine gold(I); (2) chlorotriphenylphosphine gold(I); (3) dichloro (2-pyridinecarboxylate) gold(III); and (4) 1,3-bis(2,6-diisopropylphenyl) imidazole-2-ylidene gold(I) chloride as shown in Table 1. All gold compounds were purchased from Strem Chemicals (Newburyport, MS, USA), except complex **3**, dichloro (2-pyridinecarboxylate) Au (III), which was purchased from Sigma-Aldrich, St. Louis, MO, USA. Their chemical structures are illustrated in Figure 1.

The stock solutions of the gold compounds (10 mM) were freshly prepared by dissolving the solid compound in 100% DMSO and storing the solutions at room temperature. Wherever adequate, the freshly prepared gold compound solutions were further diluted in DMSO to obtain final concentrations of 1 and/or 0.1 mM before being used.

### 2.2. Bacteria and Culture Conditions

The different bacterial strains used in this study are indicated in Table 2. Bacteria were cultivated in Brain–Heart Infusion (BHI) (Oxoid, Basingstock, UK), Luria-Bertani (LB) (Oxoid, Basingstock, UK), and Muller–Hinton (MH) (Biokar Diagnostics, Allonne, France) media. For solid and semi-solid medium (Biokar Diagnostics), agar was added at 1.5% (*w*/*v*) and 0.75% (*w*/*v*), respectively. The bacterial cells were maintained in BHI broth supplemented with 25% (*v*/*v*) glycerol at −80 °C. When required, the bacterial cells were recovered in BHI agar plates.

### 2.3. Bacteriophages

Two enterobacteria phages were used in the current study, namely Qβ DSM 13768 and Ffm DSM 18264 (Table 2). The enterobacteria phage Qβ has a positive-stranded RNA genome and is enclosed within an icosahedral capsid with a diameter of 26–27 nm. It specifically infects *E. coli* DSM 5210 and belongs to the family *Leviviridae* and to the species *Allolevivirus* (as classified by the International Committee on Taxonomy of Viruses). On the other hand, the enterobacteria phage Ffm is a lytic phage characterized by a double-stranded DNA genome. It is classified under the family *Autographiviridae* (by the International Committee on Taxonomy of Viruses) and is propagated by using *E. coli* DSM 498 [37]. The enterobacteria phages have been used to evaluate the efficacy of viral decontamination, namely respirators contaminated with viral particles [38,39,40], constituting a prompt approach to evaluate antiviral properties.

### 2.4. Antibacterial Activity

The antibacterial activity of the gold compounds was carried out by the agar diffusion technique, as previously described [34]. Briefly, each bacterial strain was first cultivated in MHagar at 37 °C for 24 h. From this culture, 3–4 colonies were transferred into 10 mL of MH broth, and the culture was incubated at 37 °C overnight. After reaching an optical density (OD_600nm_) of 0.6–1.0, 8 mL of the culture was transferred into 40 mL of liquefied MH agar medium. This mixture was then poured into a sterile Petri dish. After agar solidification, 6 mm diameter wells were created using an inverted sterile Pasteur pipette. These wells were filled with the appropriate concentrations of each gold complex, namely 100, 150, 200, 250, and 500 μM. Subsequently, the plates were incubated at 37 °C for 24 h. The inhibition zones were measured after this time interval. In this assay, both the Gram-negative bacterium *E. coli* DSM 1077 and the Gram-positive *S. aureus* ATCC 6538 were tested. Three biological and two technical replicates were performed.

#### Determination of the Minimum Inhibitory Concentration (MIC)

The Minimum Inhibitory Concentration (MIC) was determined by microdilution as previously described [34]. Briefly, the bacteria under study were grown in MH agar medium and incubated at 37 °C for 24 h. A loopful of this bacterial culture was then transferred into 10 mL of MH broth, which was subsequently incubated at 37 °C overnight with shaking (120 rpm). Next, 100 μL of MH broth containing the gold complex compounds at different concentrations (ranging from 0.95 μM to 100 μM) were distributed in each well of a 96-well flat bottom microplate (Sarstedt Inc., Nümbrecht, Germany). Each well was inoculated with 100 μL of the overnight bacterial culture. Wells containing the culture medium supplemented with DMSO or chloramphenicol (30 μg/mL) were included as controls. Additionally, a negative control consisted of bacterial growth in MH broth with no compound, while wells filled with 200 μL of MH broth were used as blanks. Three biological and three technical replicates were used. The incubation of the microplates was performed at 37 °C during 24 h. The bacterial growth was monitored by measuring the optical density at 600 nm (OD_600nm_) using a microplate reader (Tecan Infinite M200, Tecan, Austria). The MIC value was determined as the lowest concentration of the compound that caused inhibition of bacterial growth (95–100%). The lowest concentration that did not allow the recovery of the bacterial cells in MH agar plates was considered the Minimum bactericidal concentration (MBC) [34].

### 2.5. Anti-Quorum Sensing Activity

The anti-quorum Sensing (QS) properties of the gold complex **1** were evaluated using the bacterial biosensor *Chromobacterium violaceum* CV026, as previously described [41]. Briefly, *C. violaceum* CV026 was cultured on LB agar plates at 30 °C during 24 h. A loopful of the culture from each plate was then transferred to LB broth and incubated overnight in a water bath at 30 °C. Subsequently, 8 mL of this overnight culture (OD_600nm_ = 1.2) was transferred into 40 mL of liquified LB agar, followed by the addition of N-hexanoyl-homoserine lactone (C6-HSL) at a final concentration of 0.12 μg/mL. Afterward, the mixture was poured into a sterile Petri dish and allowed to solidify at room temperature inside a flow cabinet. Following solidification, wells of 6 mm were made on the solidified agar plate using an inverted sterile Pasteur pipette. These wells were filled with 40 μL of the appropriate concentration of the tested gold complex **1**. The tested concentrations were 0.07 μg/mL, 0.14 μg/mL, 0.28 μg/mL, 0.59 μg/mL, 1.14 μg/mL, and 2.32 μg/mL (0.23, 0.45, 0.91, 1.91, 3.7, and 7.51 μM). A control plate with the addition of 100 μL of phosphate-buffered solution (PBS) was included. DMSO (at its maximum concentration) served as the negative control. The plates were then incubated at 30 °C for 24 h. The absence of the violacein pigment around the well indicates inhibition of the quorum sensing system. Three biological and two technical replicates were performed.

### 2.6. Antibiofilm Activity

The ability of gold complex **1** to disrupt bacterial biofilms was evaluated following a previously described method [42] with slight modifications. Briefly, the bacteria under study were cultured in BHI agar plates at 37 °C for 24 h. After this time interval, one isolated colony was transferred into 10 mL of BHI broth and incubated at 37 °C in a water bath overnight with agitation (120 rpm). Plastic coverslips (Unbreakable; 22 × 22 mm; Fisherbrand) were distributed in 6-well flat-bottom plates (Greiner Bio-One GmbH, Kremesmunster, Austria) and were sterilized in a flow cabinet for 2 h under ultraviolet light. Subsequently, 300 μL of the overnight bacterial culture was added to 2700 μL of BHI broth, and this mixture was transferred into each well containing the coverslip. The biofilm formation was allowed to be produced at the top of the coverslips at 37 °C for 24 h. After biofilm formation, the bacterial culture was removed, and each well was washed 4 times with PBS to eliminate non-adherent cells. The biofilm slides were treated with gold (I) complex **1** for 6 h and 24 h. The biofilm slides of *S. aureus* ATCC 6538 were treated with concentrations of 1.5 μg/mL, 4.6 μg/mL, and 9 μg/mL (4.85, 14.89, and 29.12 μM, respectively), while the biofilm slides of *E. coli* DSM 1077 were treated with concentrations of 4.6 μg/mL, 9 μg/mL, and 18.5 μg/mL (14.89, 29.12, and 59.87 μM, respectively). The quantification of sessile cells (adherent) was carried out by washing the wells 4 times with PBS, and the coverslips were transferred into 10 mL of BHI broth supplemented with 0.05% Tween 80. Each tube was sonicated for 7 min at 4 °C. After sonication, the coverslip was immediately removed, and decimal serial dilutions were prepared. The viable counts were determined by the drop method [43] on BHI agar. The viable cells are expressed as Colony Forming Units by mL (CFU/mL). Controls included slides with no exposure to antibacterial agents and slides exposed to DMSO. Three biological and two technical replicates were used for each experiment.

The impact of the gold (I) compound **1** in the structure and viability of the biofilm aggregates was also evaluated by epifluorescence microscopy using the dye LIVE/DEAD BacLight Viability Kit (ThermoFisher Scientific, Waltham, MA, USA). After contact time intervals of compound **1** with the tested bacteria (6 h and 24 h), the wells were washed 4 times with PBS, and the bacterial cells were fixed with 4% (*v*/*v*) glutaraldehyde. Immediately before microscopy observations, two additional washing steps were performed, and each coverslip was mounted inverted on a microscope slide with 50 μL of LIVE/DEAD dye. The observation of the cells was performed using the microscope Axio Imager Z2 (Zeiss, Oberkochen, Germany).

### 2.7. Antiviral Activity

The antiviral activity assay was performed using the microplate method [44], followed by the double-layer agar [45]. To establish the contact between the gold compounds and bacteriophages, the gold compounds were added to the stock phage suspension at different concentrations. The tested concentrations of gold complex **1** against the Qβ phage were 0.08 mg/mL (250 μM), 0.15 mg/mL (500 μM), and 0.23 mg/mL (750 μM), while against the Ffm phage, the concentrations were 0.02 mg/mL (62.5 μM), 0.04 mg/mL (125 μM), and 0.08 mg/mL (250 μM). For gold complex **2**, the tested concentrations against the Qβ phage were 0.12 mg/mL (250 μM), 0.25 mg/mL (500 μM), and 0.37 mg/mL (750 μM), and against the Ffm phage, they were 0.03 mg/mL (62.5 μM), 0.06 mg/mL (125 μM), and 0.12 mg/mL (250 μM). The assay included controls, such as the bacterial culture in BHI broth, the bacterial culture supplemented with the compounds at different tested concentrations (to exclude any effect on the host bacterium), and controls of the host bacteria and phages (independently) supplemented with DMSO. The contact between the phage and the gold compounds was allowed for 24 h at 37 °C. After this time interval, the phage suspensions were diluted in two 96-well flat-bottom microplates (Sarstedt Inc., Nümbrecht, Germany), with each well containing 20 μL of phage/compound suspensions at different concentrations, which were then mixed with 180 μL of PBS. Sequential decimal serial dilutions were prepared in each microplate. The microplates were incubated at 37 °C, and bacterial lysis was monitored every 6 h using a microplate reader (OD_600nm_) (Tecan Infinite M200, Tecan, Austria). The last two dilutions that exhibited bacterial lysis were selected for further analysis so that the number of phage particles present in each dilution could be quantified by the double layer agar method. This information was then compared with an initial number of phages present in the initial suspension to evaluate the antiviral activity of the gold compounds.

### 2.8. Statistical Analysis

Statistical analysis was carried out using Graphpad Prism (version 9.0) (GraphPad Software, San Diego, CA, USA). Prior to analysis, normality was assessed using the Shapiro–Wilk test. To determine significant differences, one-way ANOVAs were employed, followed by Tukey’s tests for post hoc analysis, with *p*-values below 0.05 considered statistically significant.

## 3. Results

### 3.1. Antibacterial Activity

**Figure 2:**
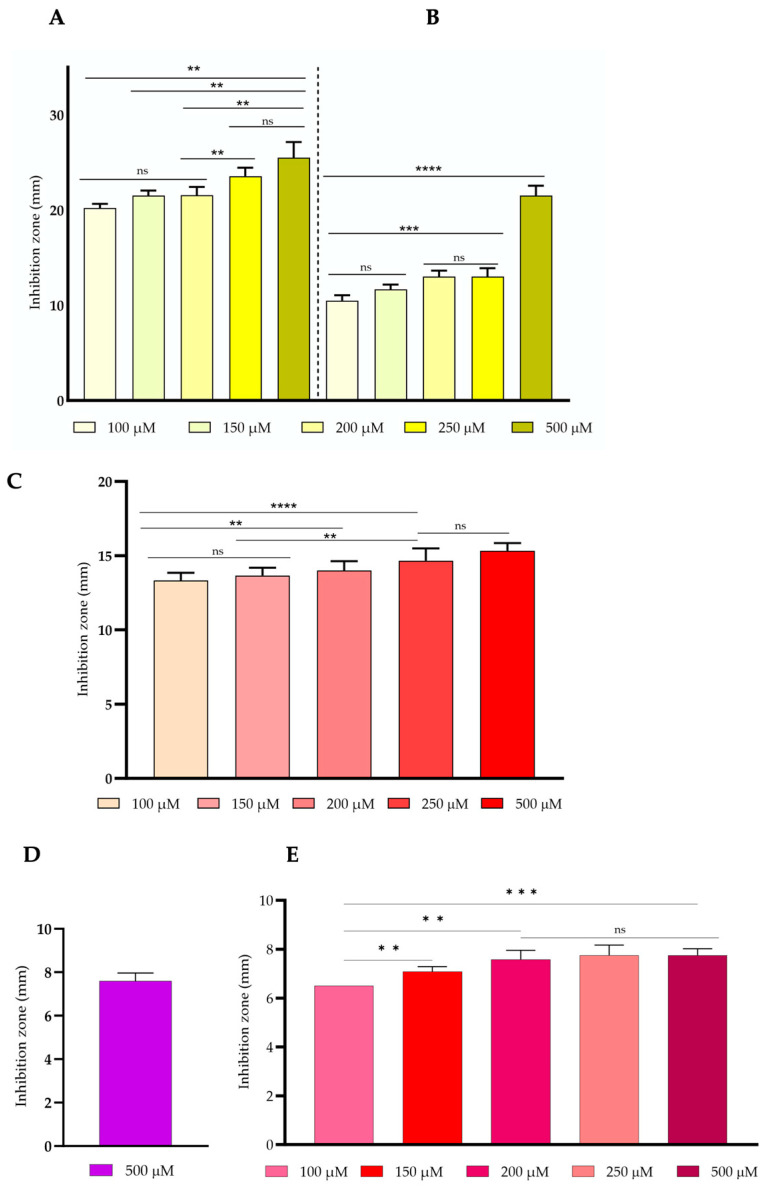
Antibacterial activity of gold (I) complex **1** (**A**) against *S. aureus* ATCC 6538; (**B**) against *E. coli* DSM 1077; (**C**) gold (I) complex **2** against *S. aureus* ATCC 6538; (**D**) gold (III) complex **3** against *S. aureus* ATCC 6538; and (**E**) gold (I) complex **4** against *S. aureus* ATCC 6538. Data are representative of three biological and two technical replicates, and error bars represent the standard deviation. ** *p* < 0.01, *** *p* < 0.001, **** *p* < 0.0001 and ns–non-significant.

The antibacterial activity of gold complexes was determined using the agar diffusion method. Gold (I) and (III) complexes were tested against both the Gram-positive bacterium, *S. aureus* ATCC 6538, and the Gram-negative *E. coli* DSM 1077. Notably, gold (I) complex **1** was able to inhibit the growth of both bacteria, while gold complexes 2, 3, and 4 were only able to inhibit the growth of *S. aureus* ATCC 6538 (Figure 2C–E). *S. aureus* ATCC 6538 was more susceptible to gold complex **1** compared to *E. coli* DSM 1077. All concentrations tested against *E. coli* DSM 1077 produced smaller inhibition zones compared to *S. aureus* ATCC 6538. For example, the lowest concentration tested (100 μM [30.9 μg/mL]) produced an inhibition zone of 19.83 ± 0.98 mm for *S. aureus* ATCC 6538, whereas the same concentration tested against *E. coli* DSM 1077 produced an inhibition zone of 10.5 ± 0.55 mm (Figure 2A,B). Notably, no significant differences (*p* > 0.05) were observed between the inhibition zones produced by the concentrations of 100 μM [30.9 μg/mL], 150 μM [46.3 μg/mL], and 200 μM [61.7 μg/mL] tested against *S. aureus* ATCC 6538. The highest concentrations tested of gold complex **1**, 250 μM [77.1 μg/mL] and 500 μM [154.34 μg/mL], against *S. aureus* ATCC 6538, produced similar inhibition zones (*p* > 0.05), namely 23.57 ± 0.89 and 25.5 ± 1.64 mm, respectively. The highest concentration of gold complex **1** tested against *E. coli* DSM 1077 resulted in an inhibition zone of 21.5 ± 1.05 mm (Figure 2B). Only *S. aureus* ATCC 6538 was susceptible to the gold (I) complex **2**, with concentrations ranging from 100 μM [49.4 μg/mL] to 200 μM [98.9 μg/mL] producing similar (*p* > 0.05) inhibition zones (13.3 ± 0.52 mm). The highest inhibition zones of gold complex **2** against *S. aureus* ATCC 6538 were observed at concentrations of 250 μM [123.7 μg/mL] and 500 μM [247.4 μg/mL], namely 14.7 ± 0.82 mm and 15.3 ± 0.52 mm, respectively (*p* > 0.05) (Figure 2C).

Regarding the antibacterial activity of gold complexes **3** and **4**, it was observed that these gold complexes were not active against *E. coli* DSM 1077, as observed for gold complex **2**. Additionally, their activity against *S. aureus* ATCC 6538 was very low. Gold complex **3** only produced an inhibition zone of 7.60 ± 0.38 mm at the highest concentration tested (500 μM, 194.95 μg/mL) (Figure 2D) and the gold complex **4** showed a similar inhibition zone (*p* > 0.05) at the highest concentrations tested 200 μM [124.22 μg/mL], 250 μM [155.28 μg/mL] and 500 μM [310.55 μg/mL], namely 7.50 ± 0.45 mm, 7.75 ± 0.42 mm and 7.75 ± 0.27 mm, respectively (Figure 2E). As expected, both bacteria were not susceptible to DMSO.

The gold (I) complex **1** showed the best antibacterial activity against the two tested bacteria, allowing us to determine its MIC and MBC values. These results were obtained for both susceptible and multi-resistant bacterial strains (Table 3). The lowest MIC and MBC value was observed for *S. aureus* ATCC 6538, with a MIC value of 0.59 μg/mL [1.91 μM] and an MBC of 4.63 μg/mL [14.98 μM], respectively. In contrast, the methicillin-resistant *S. aureus* strains, MRSA 12 and MRSA 15, exhibited a MIC value of 1.16 μg/mL [3.75 μM] and MBC value of 18.5 μg/mL [59.87 μM], respectively (approximately two times higher the MIC value displayed by the methicillin-susceptible *S. aureus* ATCC 6538) (Table 3). The MIC and MBC values exhibited by *E. coli* DSM 1077 were 4.63 μg/mL [14.98 μM] and 9.25 μg/mL [29.94 μM], respectively. However, the MIC value for the multi-resistant strain *E. coli* I731940778-1 was 9.25 μg/mL [29.94 μM], and the MBC value was 37.1 μg/mL [120.06 μM], which is approximately two times higher the MIC value and four times higher the MBC value of *E. coli* DSM 1077 (Table 3). The bacterial growth was not affected by DMSO.

### 3.2. Anti-Quorum Sensing Activity

**Figure 3:**
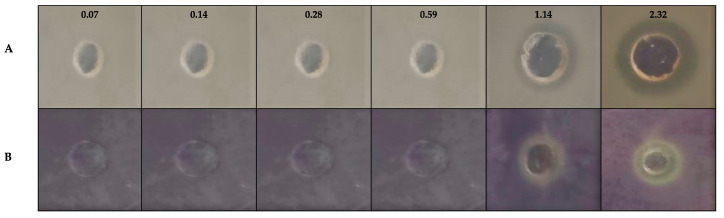
Anti-quorum sensing activity of gold (I) complex **1** using the biosensor *C. violaceum* CV026. (**A**) No addition of C6-HSL to the culture medium (control); (**B**) inhibition zone on the production of violacein (addition of C6-HS at 0.10 μg/mL) in the presence of gold (I) complex at different concentrations (μg/mL). Data are representative of three biological and two technical replicates.

The anti-quorum sensing properties of gold (I) complex **1** were evaluated using the biosensor *C. violaceum* CV026. This biosensor is an effective tool for evaluating the anti-quorum sensing activity, as it relies on the regulation of the production of the characteristic water-insoluble pigment, violacein by *C. violaceum* CV026, via the QS system [46]. A compound that inhibits the QS system of this bacterium would prevent violacein formation. In our study, the gold complex **1** was tested at different concentrations with 1.14 μg/mL [3.69 μM] and 2.32 μg/mL [7.50 μM] as the highest. Interestingly, both concentrations of gold complex **1** inhibited the production of violacein, indicating potential anti-quorum sensing activity (Figure 3). However, these concentrations also produced similar inhibition zones in the control (where no C6-HSL, a QS signal molecule, was added). This finding suggests that the inhibitory effect of gold complex **1** at these concentrations was due to the growth inhibition of the *C. violaceum* CV026 rather than directly impairing the QS system. Notably, there was no inhibition of the production of violacein, or the growth of *C. violaceum* CV026 observed in the presence of DMSO, further confirming that the observed effects were specifically attributed to gold complex **1** (Figure 3).

### 3.3. Antibiofilm Activity

**Figure 4:**
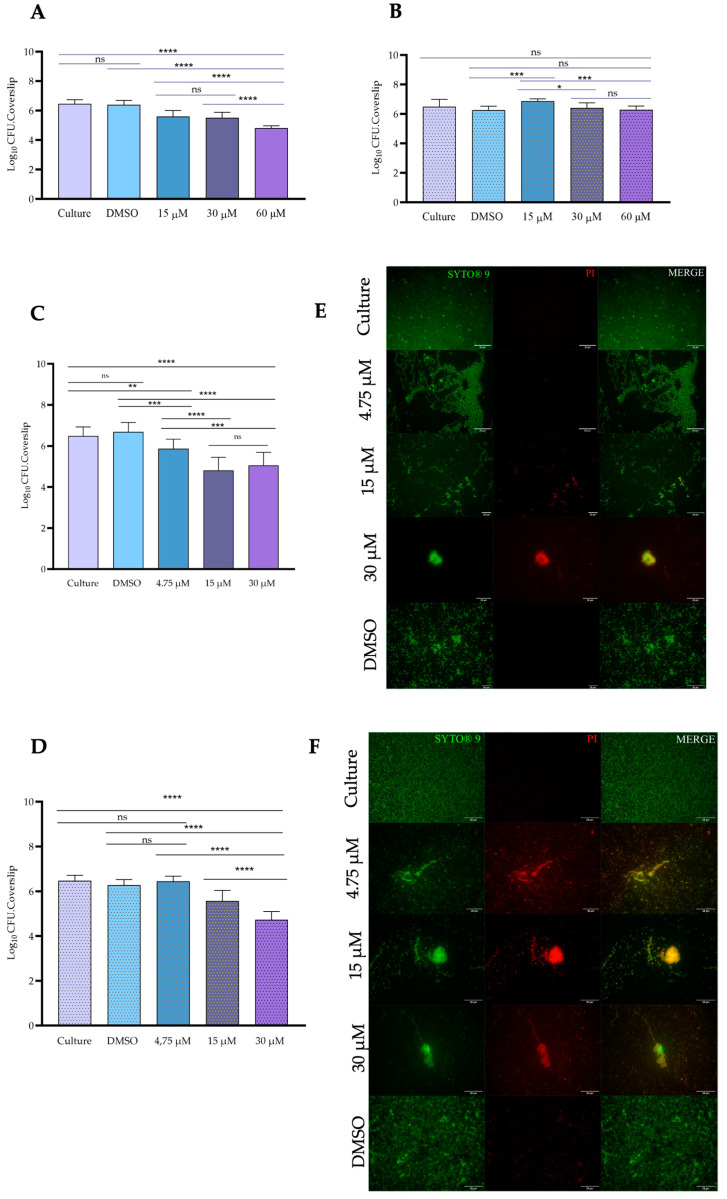
Antibiofilm activity of gold (I) complex **1** after (**A**) 6 h of treatment against the biofilm produced by *E. coli* DSM 1077; (**B**) 24 h of treatment against the biofilm produced by *E. coli* DSM 1077; (**C**) 6 h of treatment against the biofilm produced by *S. aureus* ATCC 6538; (**D**) 24 h of treatment against the biofilm produced by *S. aureus* ATCC 6538. Representative epifluorescence images of the biofilm of *S. aureus* ATCC 6538 after exposure to gold (I) complex 1 during (**E**) 6 h and (**F**) 24 h. The first column illustrates the exposure of cover slides to the dye Syto 9 (viable cells in green), the second column illustrates the stain by propidium iodide (PI) (non-viable cells in red-orange), and the third column (merged) overlaps both stains. Scale bars represent 20 μm. * *p* < 0.05, ** *p* < 0.01, *** *p* < 0.001, **** *p* < 0.0001 and ns–non-significant.

The biofilm-disrupting capacity of gold complex **1** was evaluated against the biofilms produced during 24 h by *S. aureus* ATCC 6538 and *E. coli* DSM 1077. The sessile cells were exposed to gold complex **1** for 6 h and 24 h, and after this treatment, the viable sessile cells were subsequently recovered on BHI agar plates (and expressed in Log_10_ CFU/mL). The results are illustrated in Figure 4. The exposure of sessile cells of *E. coli* DSM 1077 to gold (I) complex **1** during 6 h, at all tested concentrations, led to a significant reduction (*p* ≤ 0.0001) in comparison to controls (culture and DMSO) (Figure 4A).

Nevertheless, a significant reduction was observed at the highest concentration tested, 60 μM (18.5 μg/mL), which caused a decrease of 1.67 Log_10_. Interestingly, it was observed that the exposure of *E. coli* DSM 1077 to gold (I) complex **1** for 24 h did not impair the recovery of sessile cells, evidenced by a similar (*p* > 0.05) recovery between treated and non-treated sessile cells (control). Intriguingly, the recovery of sessile cells exposed to the lowest concentration, 15 μM (4.6 μg/mL), was higher (*p* < 0.001) in comparison to the control cells (culture) (Figure 4B).

A disturbance of the biofilm produced by *S. aureus* ATCC 6538 by exposure to gold (I) complex **1** during 6 h was observed for all concentrations tested (Figure 4C). The concentrations of 15 μM (4.6 μg/mL) and 30 μM (9.0 μg/mL) equally (*p* > 0.05) impaired the recovery of sessile cells, resulting in a decrease of 1.59 Log_10_. In contrast with *E. coli* DSM 1077, the extended exposure of the biofilm formed by *S. aureus* ATCC 6538 during 24 h resulted in a significant (*p* < 0.0001) decline in the viability of the sessile cells treated with 15 μM and 30 μM (4.6 μg/mL and 9.0 μg/mL), namely 0.91 and 1.5 Log_10_, respectively (Figure 4D). The gold complex (I) **1**, at a concentration of 4.75 μM (1.47 μg/mL), was able to impair the recovery of sessile cells of *S. aureus* ATCC 6538 after 6 h, but no impact (*p* > 0.05) was observed after 24 h (Figure 4C,D). The exposure of the biofilm to DMSO did not affect the recovery of sessile cells for both *E. coli* DSM 1077 and *S. aureus* ATCC 6538 (Figure 4A–D).

The impact of the exposure of the biofilm produced by *S. aureus* ATCC 6538 to gold complex (I) **1**, during 6 h and 24 h, was also investigated by epifluorescence microscopy using the LIVE/ DEAD Bac/Light dye. The results are depicted in Figure 4E,F. It is possible to observe that after 6 h of exposure to gold complex **1**, the structure of the biofilm is notably disrupted, particularly at the lowest concentration, 4.75 μM (1.47 μg/mL), where clear zones without sessile cells were noticed, but most cells remained viable (Figure 4E). The disruption of the biofilm became more pronounced at an intermediate concentration of 15 μM (4.6 μg/mL), with sessile cells exhibiting compromised viability (appearing orange). At the highest concentration tested, 30 μM (9.0 μg/mL), only small aggregates and dispersed cells with compromised viability (appearing orange-red) were observed, indicating a substantial disruption of the biofilm structure (Figure 4E). Following the extended exposure of the biofilm to gold complex **1** during 24 h, a similar disruption pattern was observed on the biofilm, as seen at 6 h. The biofilm structure was disturbed, and the viability of sessile cells was more markedly impaired at the higher concentrations of 15 μM and 30 μM (4.6 μg/mL and 9.0 μg/mL). In contrast, exposure of the biofilm of *S. aureus* ATCC 6538 to DMSO during 6 h or 24 h did not cause any disorder in the biofilm structure or compromise the viability of sessile cells (Figure 4C–F).

### 3.4. Antiviral Activity

**Figure 5:**
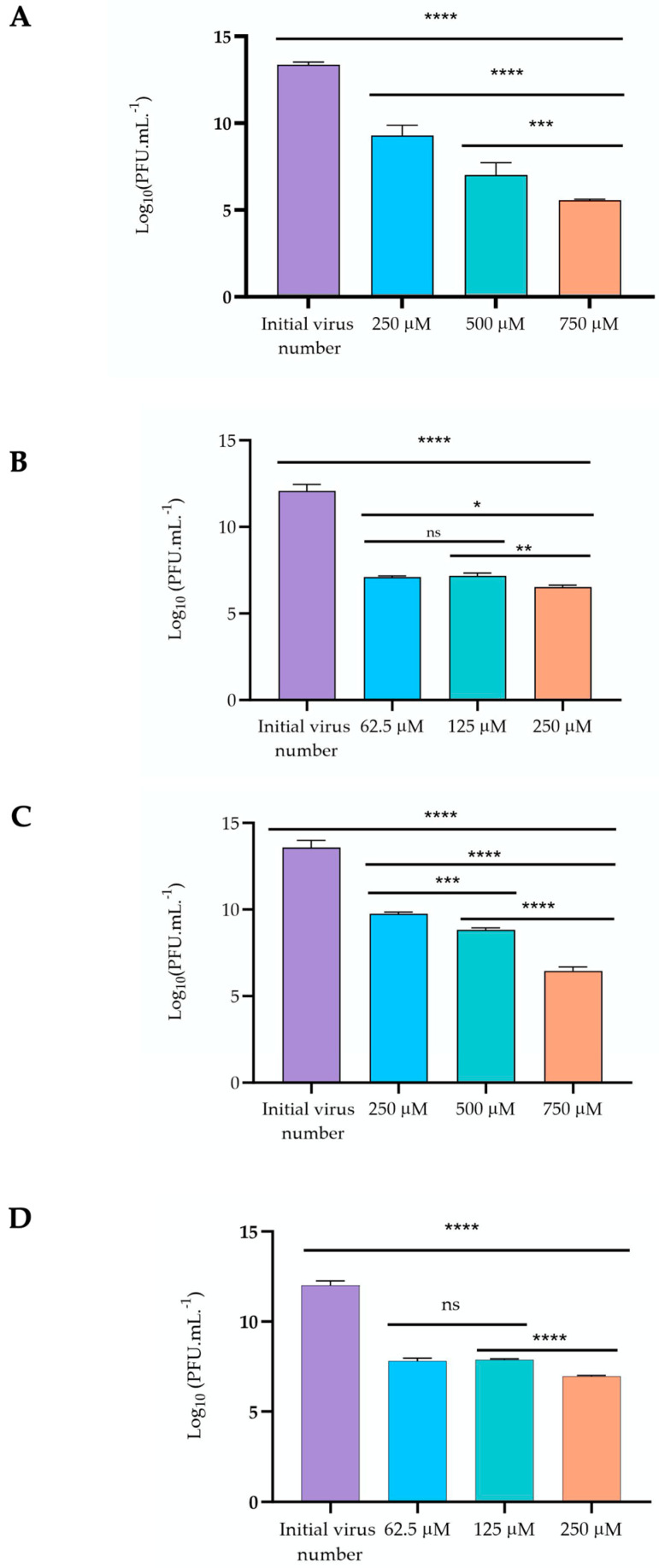
Antiviral activity of gold (I) complexes **1** and **2** against bacteriophage Qβ and Ffm: (**A**) Gold (I) complex **1** against bacteriophage Qβ; (**B**) Gold (I) complex **1** against bacteriophage Ffm; (**C**) Gold (I) complex **2** against bacteriophage Qβ (**D**); Gold (I) complex **2** against bacteriophage Ffm. The number of phage plaques (Log_10_ PFU/mL) was determined after 24 h of incubation and compared with the initial virus number. Data are the mean of three independent experiments, and error bars represent the standard deviation. Significant differences between the number of phage plaques are indicated, * *p* < 0.05, ** *p* < 0.01, *** *p* < 0.001, **** *p* < 0.0001 and ns–non-significant.

The antiviral activity of gold (I) complexes **1** and **2** was assessed against two different bacteriophages, namely the bacteriophage Ffm, which is a double-stranded DNA phage, and the bacteriophage Qβ, which is a single-stranded RNA phage. Both bacteriophages were exposed to different concentrations of the gold (I) complexes **1** and **2** for 24 h, and the virus plaques were subsequently quantified. The results are expressed as Log_10_ PFU/mL, as depicted in Figure 5.

The susceptibility of the bacteriophage Qβ follows a concentration-dependent pattern for both gold (I) complexes (**1** and **2**); however, it is more susceptible to gold (I) complex **1** than to gold (I) complex **2**.

The gold (I) complex **1** at the highest concentration tested, 750μM (231 μg/mL), caused a remarkable reduction in virus plaques of 7.81 Log (Figure 5A). In comparison, a reduction of 7.16 Log was found for gold (I) complex **2** at the highest concentration tested at 750 μM (371.1μg/mL) (Figure 5C).

Similarly, bacteriophage Ffm was also more susceptible to gold (I) complex **1** than to gold (I) complex **2** (Figure 5B,D). The concentrations of gold (I) complex **1** of 62.5 μM (19.31 μg/mL) and 125 μM (38.62 μg/mL) against bacteriophage Ffm, showed similar activity (*p* > 0.05), followed by the reduction in virus plaques (*p* < 0.05) for the highest concentration 250 μM (Figure 5B). In contrast, all concentrations of gold (I) complex **2** significantly reduced (*p* < 0.0001) the virus plaque count of Ffm (Figure 5D). The lower concentrations of 62.5 μM (30.89 μg/mL) and 125 μM (61.75 μg/mL) caused similar reductions (*p* > 0.05) in the counts of the virus plaque of Ffm, whereas the highest concentration of 250 μM (123.70 μg/mL) caused the highest reduction (*p* < 0.0001), amounting to 5.04 Log_10_ (Figure 5D).

## 4. Discussion

### 4.1. Antibacterial Activity

In the current study, the antibacterial, antibiofilm, anti-quorum sensing, and antiviral activities of four gold (I and III) complexes were evaluated. Among them, gold complex (I) **1** showed a better performance than the others. The MIC value of the gold complex (I) **1** against the Gram-positive *S. aureus* ATCC 6538 was much lower than the reported value for the well-known gold complex Auranofin [47]. In the study conducted by Cassetta et al. [47], the MIC values of Auranofin for *S. aureus* ATCC 25923 and *S. aureus* USA 300 varied between 250 and 500 μg/mL, whereas the reported value for the gold complex (I) **1** in our current study was only 0.59 μg/mL for *S. aureus* ATCC 6538. Similarly, the MIC values of Auranofin reported by Cassetta et al. [47] for MRSA strains varied between 125 and 250 μg/mL, in contrast to gold complex (I) **1** that displayed a MIC value of 1.16 μg/mL for MRSA strains. In the study of Schmidt et al. [48], two MRSA strains were affected by the exposure to gold (I) N-heterocyclic carbene complex with MIC values quite similar to those obtained in our current study, namely 0.28 μg/mL. Moreover, the gold complex (I) **1** demonstrated effective action against Gram-negative bacteria, indicating its ability to control the growth of these typically more challenging bacteria. The MIC value of gold complex (I) **1** against *E. coli* DSM 1077 was considerably lower than that reported for Auranofin against the strain *E. coli* ATCC 25922 (>8000 μg/mL). Additionally, the gold complex (I) **1** was also effective against the multi-resistant *E. coli* I731940778-1. It is important to highlight that bacterial strains can largely vary in their susceptibility to antibacterial agents. For this reason, it is always advisable to include a panel of strains in such studies, as we did in our study and as conducted in other studies [17,34,47], to comprehensively evaluate the efficacy and potential of such antibacterial agents.

The antibacterial activity of gold (I) complexes has been attributed to their ability to induce alterations of the bacterial surface, resulting in the leaking of the cytoplasmic content. This is probably due to the injury caused on the peptidoglycan layer when the gold complexes penetrate the bacterial lipid bilayer, thereby compromising the structural integrity of the bacterial cell [49,50,51]. However, the lower antibacterial activity of gold (I) complexes against the Gram-negative *E. coli* is likely due to their limited capacity to overcome the protective outer membrane of these bacteria [52].

The bacterial cells in the adherent state, forming sessile cells either on biotic or abiotic surfaces, generally display reduced susceptibility to various antibacterial agents in comparison to their planktonic counterparts [8]. Our findings, together with those reported by other authors [51,53,54,55], provide evidence of the potential of gold complexes as antibiofilm agents. Specifically, the gold complex (I) **1** was able to disrupt biofilm formation by *S. aureus* ATCC 6538 and *E. coli* DSM 1077, showing greater efficiency against the biofilm produced by *S. aureus* ATCC 6538 after 6 h or 24 h of treatment, particularly at concentrations of 15 μM and 30 μM (4.6 μg/mL and 9.0 μg/mL). The study of Samanta et al. [51] reported that the N,N′-olefin functionalized bis-imidazolium gold (I) salt successfully impaired the biofilm formed by a clinical isolate of *S. aureus* on contact lenses, namely at a concentration of 3.90 μM. In a study conducted by Torres et al. [55], it was reported that auranofin at 7.94 μg/mL (11.7 μM) was able to inhibit 50% of the biofilm formed by the MRSA strain TCH1516. Although the mechanism of action of gold complexes against bacterial biofilms has not yet been clarified, these compounds likely need to first penetrate the complex matrix of exopolymeric substances that coat sessile cells and then directly act on the bacterial cells. Further studies are required to fully elucidate this activity since the use of gold complexes to control infections related to medical devices, implants, wounds, and burns could be an important tool.

### 4.2. Anti-Quorum Sensing and Antibiofilm Activity

The evaluation of the anti-quorum sensing (QS) activity of gold complex (I) **1** using the biosensor *C. violaceum* CV026 revealed inhibition of violacein production. However, it was also observed that this compound affected the growth of the biosensor, making it difficult to conclusively attribute the reduction in violacein production only to interference with the QS system of this bacterium. Nevertheless, it is plausible that gold complex (I) **1** may indeed affect the QS of other Gram-negative bacteria, such as *E. coli* since it was observed an impairment on biofilm formation in *E. coli* DSM 1077.

The biofilm formation in *E. coli* involves the autoinducer AI-2 (furanosyl borate diester) encoded by the gene *luxS* (S-ribosylhomocysteine), which induces biofilm formation and regulates the biofilm structure [56]. On the other hand, the QS system in Gram-positive bacteria, like *S. aureus*, involves different autoinducer molecules, known as oligopeptides, instead of homoserine lactones. In *S. aureus,* the QS system is controlled by the staphylococcal accessory regulator (*sar*) and the accessory gene regulator (*agr*) in a cascade. The *agr* system produces oligopeptides as signaling molecules that regulate the expression of secreted virulence factors and are also involved in the dispersal of biofilms [57]. Likewise, the *sar* system acts as a global control of the QS cascade, regulating virulence and biofilm production [58]. Considering that gold complex **1** was able to affect the biofilm formed in both *E. coli* and *S. aureus*, we anticipate that in *E. coli*, the gold complex (I) **1** may interfere with the expression of the *luxS* gene or the degradation of AI-2. In *S. aureus*, both *agr* and *sar* systems, or each individually, may be impaired by the gold complex **1**. Although our findings suggest the potential of gold complex (I) **1** to interfere with QS systems in *E. coli* and *S. aureus*, further studies are required to confirm these hypotheses.

### 4.3. Antiviral Activity

Regarding the antiviral activity of gold complexes **1** and **2**, it was evident that both compounds were capable of affecting the two tested phages, with gold complex **1** exhibiting better antiviral activity. The investigation of the antiviral activity of gold complexes remains limited, but some studies have explored their potential [59,60,61,62]. For instance, triphenylphosphine gold (I) derivatives displayed different actions against the Chikungunya virus (CHIKV), with the compound AuCl(PPh_3_) being able to affect the replication of the virus by 99%. This effect can be attributed to its interaction with dsRNA [62]. Furthermore, the action of auranofin and other gold organometallics against the severe acute respiratory syndrome coronavirus 2 (SARS-CoV-2) has been evaluated [60,61,62], revealing interactions with the papain-like protease (PLpro) and the receptor binding domain (RBD) of the S1 subunit of the (SARS-CoV-2). These interactions have demonstrated promising IC_50_ values at very low concentrations (1.0 μM) and suitable IC_50_ values (16–25 μM) [59]. In the case of the enterovirus, it is conceivable that gold complexes **1** and **2** may exert their antiviral effects by interfering with the dsDNA and RNA, thereby limiting the replication of the virus.

## 5. Conclusions

In conclusion, the results of the current study highlight the significant antibacterial activity (including against multi-resistant bacteria), antibiofilm, and antiviral activities of the gold complex **1**. Although this complex did not exhibit anti-quorum sensing activity using the biosensor *C. violaceum* CV026, it demonstrated antibiofilm activity against both *E. coli* and *S. aureus*. This suggests that it may disrupt quorum sensing activity in both bacteria, given that the biofilm formation in both is regulated by quorum sensing elements. This hypothesis will be further investigated and tested in subsequent studies. Overall, our results emphasize the potential use of chlorotrimethylphosphinegold (I) **1** as a promising candidate for controlling both bacterial and viral infections.

## Figures and Tables

**Table 1:** Gold compounds used in the study.

Formula	Abbreviation	Net Charge	MW (g/mol)	CAS Number	Purity
C_3_H_9_PAuCl	1	+1	308.50	15278-97-4	99%
C_18_H_15_PAuCl	2	+1	494.71	14243-64-2	≥99.9%
C_6_H_4_NAuCl_2_O_2_	3	+3	389.97	88215-41-2	99%
C_27_H_36_AuClN_2_	4	+1	621.01	852445-83-1	99%

**Table 2:** Microorganisms used in the study.

Bacteria	Origin and Characteristics	Source
*Escherichia coli* DSM 1077	K12 galR arg nad	German Collection of Microorganisms
*Escherichia coli* DSM 5210	Hfr 3000 U 432. Host of phage Qß (DSM 5696)	German Collection of Microorganisms
*Escherichia coli* DSM 498	K12 wildtype. Host of phage Ffm (DSM 18264)	German Collection of Microorganisms
*Escherichia coli* I731940778-1	Multi-resistant. Isolated from urine	Laboratory of Microbiology, ABC-RI, UAlg ^1^
*Staphylococcus aureus* ATCC 6538	Wound	American Type Culture Collection
*Staphylococcus aureus* methicillin-resistant 15 (MRSA 15)	Clinic	Laboratory of Microbiology, ABC-RI, UAlg ^1^
*Staphylococcus aureus* methicillin-resistant 16 (MRSA 16)	Clinic	Laboratory of Microbiology, ABC-RI, UAlg ^1^
*Chromobacterium violaceum* (CV026)	Biosensor strain of the production of homoserine lactone (HgR, cvil::Tn5 xylE, KanR, higher spontaneous resistance StrR)	Gift of Professor Mondher El Jaziri of the University Libre of Brussels
Bacteriophages	Family	Host
*Escherichia coli* phage Ffm DSM 18264	*Autographiviridae*	For propagation, *E. coli* DSM 498, rough-LPS *Salmonella* mutants are also receptors
*Escherichia coli* phage Qbeta (Enterobacteria phage Qbeta) DSM 13768	*Leviviridae*	For propagation *E. coli* DSM 5210

^1^ UAlg-Universidade do Algarve.

**Table 3:** Minimum Inhibitory Concentration (MIC) and Minimum Bactericidal Concentration (MBC) values of gold (I) complex **1** for the tested bacteria.

Bacteria	MIC (μg/mL) ^1^	MBC (μg/mL) ^1^
*S. aureus* ATCC 6538	0.59	4.63
MRSA12	1.16	18.5
MRSA15	1.16	18.5
*E. coli* DSM 1077	4.63	9.25
Multi-resistant *E. coli* I731940778-1	9.25	37.1

^1^ Data are representative of three biological and two technical replicates.

## Data Availability

The data presented in the current study are available within the article.

## References

[1] Murray C.J., Ikuta K.S., Sharara F., Swetschinski L., Robles Aguilar G., Gray A., Han C., Bisignano C., Rao P., Wool E. (2022). Global burden of bacterial antimicrobial resistance in 2019: A systematic analysis. Lancet.

[2] Apolónio J., Faleiro M.L., Miguel M.G., Neto L. (2014). No induction of antimicrobial resistance in Staphylococcus aureus and Listeria monocytogenes during continuous exposure to eugenol and citral. FEMS Microbiol. Lett..

[3] Mashayamombe M., Carda-Diéguez M., Mira A., Fitridge R., Zilm P.S., Kidd S.P. (2023). Subpopulations in Strains of Staphylococcus aureus Provide Antibiotic Tolerance. Antibiotics.

[4] Tiedje J.M., Fu Y., Mei Z., Schäffer A., Dou Q., Amelung W., Elsner M., Adu-Gyamfi J., Heng L., Virta M. (2023). Antibiotic resistance genes in food production systems support One Health opinions. Curr. Opin. Environ. Sci. Health.

[5] Hegemann J.D., Birkelbach J., Walesch S., Müller R. (2022). Current developments in antibiotic discovery: Global microbial diversity as a source for evolutionary optimized anti-bacterials. EMBO Rep..

[6] Walesch S., Birkelbach J., Jézéquel G., Haeckl F.P.J., Hegemann J.D., Hesterkamp T., Hirsch A.K.H., Hammann P., Müller R. (2023). Fighting antibiotic resistance—Strategies and (pre)clinical developments to find new antibacterials. EMBO Rep..

[7] Mancuso G., Midiri A., Gerace E., Biondo C. (2021). Bacterial Antibiotic Resistance: The Most Critical Pathogens. Pathogens.

[8] Uruén C., Chopo-Escuin G., Tommassen J., Mainar-Jaime R.C., Arenas J. (2021). Biofilms as promoters of bacterial antibiotic resistance and tolerance. Antibiotics.

[9] Caldara M., Belgiovine C., Secchi E., Rusconi R. (2022). Environmental, Microbiological, and Immunological Features of Bacterial Biofilms Associated with Implanted Medical Devices. Clin. Microbiol. Rev..

[10] Rumbaugh K.P., Sauer K. (2020). Biofilm dispersion. Nat. Rev. Microbiol..

[11] Flemming H.-C., van Hullebusch E.D., Neu T.R., Nielsen P.H., Seviour T., Stoodley P., Wingender J., Wuertz S. (2023). The biofilm matrix: Multitasking in a shared space. Nat. Rev. Microbiol..

[12] Mukherjee S., Bassler B.L. (2019). Bacterial quorum sensing in complex and dynamically changing environments. Nat. Rev. Microbiol..

[13] Wang Y., Tian T., Zhang J., Jin X., Yue H., Zhang X.H., Du L., Bai F. (2019). Indole reverses intrinsic antibiotic resistance by activating a novel dual-function importer. MBio.

[14] Wang Y., Bian Z., Wang Y. (2022). Biofilm formation and inhibition mediated by bacterial quorum sensing. Appl. Microbiol. Biotechnol..

[15] Alavi M., Li L., Nokhodchi A. (2023). Metal, metal oxide and polymeric nanoformulations for the inhibition of bacterial quorum sensing. Drug Discov. Today.

[16] Sionov R.V., Steinberg D. (2022). Targeting the Holy Triangle of Quorum Sensing, Biofilm Formation, and Antibiotic Resistance in Pathogenic Bacteria. Microorganisms.

[17] Marzo T., Cirri D., Pollini S., Prato M., Fallani S., Cassetta M.I., Novelli A., Rossolini G.M., Messori L. (2018). Auranofin and its Analogues Show Potent Antimicrobial Activity against Multidrug-Resistant Pathogens: Structure–Activity Relationships. ChemMedChem.

[18] Büssing R., Karge B., Lippmann P., Jones P.G., Brönstrup M., Ott I. (2021). Gold(I) and Gold(III) N-Heterocyclic Carbene Complexes as Antibacterial Agents and Inhibitors of Bacterial Thioredoxin Reductase. ChemMedChem.

[19] Chakraborty P., Oosterhuis D., Bonsignore R., Casini A., Olinga P., Scheffers D.J. (2021). An Organogold Compound as Potential Antimicrobial Agent against Drug-Resistant Bacteria: Initial Mechanistic Insights. ChemMedChem.

[20] Ratia C., Cepas V., Soengas R., Navarro Y., Velasco-de Andrés M., Iglesias M.J., Lozano F., López-Ortiz F., Soto S.M. (2022). A C∧S-Cyclometallated Gold(III) Complex as a Novel Antibacterial Candidate Against Drug-Resistant Bacteria. Front. Microbiol..

[21] Chen X., Sun S., Huang S., Yang H., Ye Q., Lv L., Liang Y., Shan J., Xu J., Liu W. (2023). Gold(I) selenium N-heterocyclic carbene complexes as potent antibacterial agents against multidrug-resistant gram-negative bacteria via inhibiting thioredoxin reductase. Redox Biol..

[22] Radzig M., Koksharova O., Khmel I., Ivanov V., Yorov K., Kiwi J., Rtimi S., Tastekova E., Aybush A., Nadtochenko V. (2019). Femtosecond spectroscopy of au hot-electron injection into tio 2: Evidence for au/tio 2 plasmon photocatalysis by bactericidal au ions and related phenomena. Nanomaterials.

[23] Dominelli B., Correia J.D.G., Kühn F.E. (2018). Medicinal Applications of Gold(I/III)-Based Complexes Bearing N-Heterocyclic Carbene and Phosphine Ligands. J. Organomet. Chem..

[24] Balfourier A., Kolosnjaj-Tabi J., Luciani N., Carn F., Gazeau F., Murphy C.J. (2020). Gold-based therapy: From past to present. Proc. Natl. Acad. Sci. USA.

[25] Forestier J. (1932). The treatment of rheumatoid arthritis with gold salts injections. Lancet.

[26] Hornos Carneiro M.F., Barbosa F. (2016). Gold nanoparticles: A critical review of therapeutic applications and toxicological aspects. J. Toxicol. Environ. Health Part B Crit. Rev..

[27] Yao L., Bojic D., Liu M. (2023). Applications and safety of gold nanoparticles as therapeutic devices in clinical trials. J. Pharm. Anal..

[28] Carabineiro S.A.C., Jarnagin L.H.A. (2013). Synthesis and Applications of Gold Nanoparticles. Gold Nanoparticles: Synthesis, Optical Properties and Applications for Cancer Treatment.

[29] Higby G. (1982). Gold in Medicine-A review of its use in the West before 1900. Gold Bull..

[30] Carabineiro S.A.C. (2017). Applications of gold nanoparticles in nanomedicine-Recent advances in vaccines. Molecules.

[31] Berrocal M., Cordoba-Granados J.J., Carabineiro S.A.C., Gutierrez-Merino C., Aureliano M., Mata A.M. (2021). Gold compounds inhibit the ca2+-atpase activity of brain pmca and human neuroblastoma sh-sy5y cells and decrease cell viability. Metals.

[32] Fonseca C., Fraqueza G., Carabineiro S.A.C., Aureliano M. (2020). The ca2+-atpase inhibition potential of gold(I, iii) compounds. Inorganics.

[33] Aureliano M., Marques-da-Silva D., Serrano A., Martins J., Faleiro L., Fonseca C., Fraqueza G., Lagoa R., Rubio L.R., Vilela J.L.V., Artetxe B., Gutiérrez-Zorrilla J.M. (2023). Polyoxometalates with anticancer, antibacterial and antiviral activities. Polyoxometalates: Advances, Properties, and Applications.

[34] Faleiro L., Marques A., Martins J., Jordão L., Nogueira I., Gumerova N.I., Rompel A., Aureliano M. (2022). The Preyssler-Type Polyoxotungstate Exhibits Anti-Quorum Sensing, Antibiofilm, and Antiviral Activities. Biology.

[35] Aureliano M., Gumerova N.I., Sciortino G., Garribba E., Rompel A., Crans D.C. (2021). Polyoxovanadates with emerging biomedical activities. Coord. Chem. Rev..

[36] Aureliano M., De Sousa-Coelho A.L., Dolan C.C., Roess D.A., Crans D.C. (2023). Biological Consequences of Vanadium Effects on Formation of Reactive Oxygen Species and Lipid Peroxidation. Int. J. Mol. Sci..

[37] Adler B.A., Kazakov A.E., Zhong C., Liu H., Kutter E., Lui L.M., Nielsen T.N., Carion H., Deutschbauer A.M., Mutalik V.K. (2021). The genetic basis of phage susceptibility, cross-resistance and host-range in Salmonella. Microbiology.

[38] Vo E., Rengasamy S., Shaffer R. (2009). Development of a test system to evaluate procedures for decontamination of respirators containing viral droplets. Appl. Environ. Microbiol..

[39] Boudaud N., Machinal C., David F., Fréval-Le Bourdonnec A., Jossent J., Bakanga F., Arnal C., Jaffrezic M.P., Oberti S., Gantzer C. (2012). Removal of MS2, Qβ and GA bacteriophages during drinking water treatment at pilot scale. Water Res..

[40] Brady T.M., Strauch A.L., Almaguer C.M., Niezgoda G., Shaffer R.E., Yorio P.L., Fisher E.M. (2017). Transfer of bacteriophage MS2 and fluorescein from N95 filtering facepiece respirators to hands: Measuring fomite potential. J. Occup. Environ. Hyg..

[41] El-Guendouz S., Aazza S., Lyoussi B., Bankova V., Popova M., Neto L., Faleiro M.L., Da Graça Miguel M. (2018). Moroccan Propolis: A Natural Antioxidant, Antibacterial, and Antibiofilm against Staphylococcus aureus with No Induction of Resistance after Continuous Exposure. Evid. Based Complement. Altern. Med..

[42] Walker J.N., Horswill A.R. (2012). A coverslip-based technique for evaluating Staphylococcus aureus biofilm formation on human plasma. Front. Cell. Infect. Microbiol..

[43] Miles A.A., Misra S.S., Irwin J.O. (1938). The estimation of the bactericidal power of the blood. J. Hyg..

[44] McLaughlin M.R. (2007). Simple colorimetric microplate test of phage lysis in Salmonella enterica. J. Microbiol. Methods.

[45] Miguel M.G., Faleiro L., Antunes M.D., Aazza S., Duarte J., Silvério A.R. (2013). Antimicrobial, antiviral and antioxidant activities of “ água-mel” from Portugal. Food Chem. Toxicol..

[46] McClean K.H., Winson M.K., Fish L., Taylor A., Chhabra S.R., Camara M., Daykin M., Lamb J.H., Swift S., Bycroft B.W. (1997). Quorum sensing and Chromobacterium violaceum: Exploitation of violacein production and inhibition for the detection of N-acylhomoserine lactones. Microbiology.

[47] Cassetta M.I., Marzo T., Fallani S., Novelli A., Messori L. (2014). Drug repositioning: Auranofin as a prospective antimicrobial agent for the treatment of severe staphylococcal infections. BioMetals.

[48] Schmidt C., Karge B., Misgeld R., Prokop A., Franke R., Brönstrup M., Ott I. (2017). Gold(I) NHC Complexes: Antiproliferative Activity, Cellular Uptake, Inhibition of Mammalian and Bacterial Thioredoxin Reductases, and Gram-Positive Directed Antibacterial Effects. Chem. A Eur. J..

[49] Ratia C., Soengas R.G., Soto S.M. (2022). Gold-Derived Molecules as New Antimicrobial Agents. Front. Microbiol..

[50] Torres M.R., Slate A.J., Ryder S.F., Akram M., Iruzubieta C.J.C., Whitehead K.A. (2021). Ionic gold demonstrates antimicrobial activity against Pseudomonas aeruginosa strains due to cellular ultrastructure damage. Arch. Microbiol..

[51] Samanta T., Roymahapatra G., Porto W.F., Seth S., Ghorai S., Saha S., Sengupta J., Franco O.L., Dinda J., Mandal S.M. (2013). N, N′-Olefin Functionalized Bis-Imidazolium Gold(I) Salt Is an Efficient Candidate to Control Keratitis-Associated Eye Infection. PLoS ONE.

[52] Torres N.S., Montelongo-Jauregui D., Abercrombie J.J., Srinivasan A., Lopez-Ribot J.L., Ramasubramanian A.K., Leung K.P. (2018). Antimicrobial and antibiofilm activity of synergistic combinations of a commercially available small compound library with colistin against Pseudomonas aeruginosa. Front. Microbiol..

[53] Aragoni M.C., Podda E., Caria V., Carta S.A., Cherchi M.F., Lippolis V., Murgia S., Orrù G., Pippia G., Scano A. (2023). [AuIII(N^N)Br2](PF6): A Class of Antibacterial and Antibiofilm Complexes (N^N = 2,2′-Bipyridine and 1,10-Phenanthroline Derivatives). Inorg. Chem..

[54] Ratia C., Ballén V., Gabasa Y., Soengas R.G., Velasco-de Andrés M., Iglesias M.J., Cheng Q., Lozano F., Arnér E.S.J., López-Ortiz F. (2023). Novel gold(III)-dithiocarbamate complex targeting bacterial thioredoxin reductase: Antimicrobial activity, synergy, toxicity, and mechanistic insights. Front. Microbiol..

[55] Torres N.S., Abercrombie J.J., Srinivasan A., Lopez-Ribot J.L., Ramasubramanian A.K., Leung K.P. (2016). Screening a commercial library of pharmacologically active small molecules against Staphylococcus aureus biofilms. Antimicrob. Agents Chemother..

[56] Sharma G., Sharma S., Sharma P., Chandola D., Dang S., Gupta S., Gabrani R. (2016). Escherichia coli biofilm: Development and therapeutic strategies. J. Appl. Microbiol..

[57] Boles B.R., Horswill A.R. (2008). agr-Mediated Dispersal of Staphylococcus aureus biofilms. PLoS Pathog..

[58] Pratten J., Foster S.J., Chan P.F., Wilson M., Nair S.P. (2001). Staphylococcus aureus accessory regulators: Expression within biofilms and effect on adhesion. Microbes Infect..

[59] Gil-Moles M., Basu U., Büssing R., Hoffmeister H., Türck S., Varchmin A., Ott I. (2020). Gold Metallodrugs to Target Coronavirus Proteins: Inhibitory Effects on the Spike-ACE2 Interaction and on PLpro Protease Activity by Auranofin and Gold Organometallics. Chem. A Eur. J..

[60] Marzo T., Messori L. (2020). A Role for Metal-Based Drugs in Fighting COVID-19 Infection? The Case of Auranofin. ACS Med. Chem. Lett..

[61] Sonzogni-Desautels K., Ndao M. (2021). Will Auranofin Become a Golden New Treatment Against COVID-19?. Front. Immunol..

[62] Aires R.L., Santos I.A., Fontes J.V., Bergamini F.R.G., Jardim A.C.G., Abbehausen C. (2022). Triphenylphosphine gold (I) derivatives promote antiviral effects against the Chikungunya virus. Metallomics.

